# Caught in the undertow: a qualitative study exploring the relationship between the sustainable employability of healthcare workers and quality of care

**DOI:** 10.1136/bmjopen-2025-108470

**Published:** 2025-12-14

**Authors:** Iris van de Voort, Ian Leistikow, Roland Bal, Jan-Willem Weenink

**Affiliations:** 1 Erasmus School of Health Policy and Management, Erasmus University Rotterdam, Rotterdam, The Netherlands; 2 Health and Youth Care Inspectorate, Utrecht, The Netherlands

**Keywords:** Quality in health care, Quality Improvement, Hospitals, Health Workforce, QUALITATIVE RESEARCH

## Abstract

**Abstract:**

**Objectives:**

The sustainable employability of healthcare workers is associated with quality of care and vice versa, but how both interact remains largely unknown. This study aims to better understand the underlying mechanisms that explain the interconnectedness between healthcare workers’ sustainable employability and quality of care by examining organisational practices in two hospital teams that work on improving specific clinical processes.

**Design:**

A qualitative study was conducted, where team leaders, department managers and healthcare teams were observed and interviewed about their experiences with, and perspectives on, the (organisation of the) respective clinical process and daily (quality improvement) work. Transcripts and field notes were analysed in accordance with reflexive thematic analysis.

**Setting:**

The emergency room and operating room of a recently merged Dutch hospital.

**Participants:**

A total of 49 hours of observations and 10 interviews were conducted with team leaders, department managers, (scrub) nurses, physicians and other allied health professionals. Interviewees were purposively recruited when they were involved in, or considered knowledgeable about, the clinical processes.

**Analysis:**

This study identified three mechanisms as a result of different organisational practices that affected healthcare workers’ sustainable employability and quality of care separately and set in motion their interconnectedness: routinely overburdened staff, prolonged perceived distance between staff and regular disregard of raised concerns by staff. Over time, as these mechanisms remained unaddressed, undertows of slumbering sentiments—discontent, distrust and inertia—emerged. These sentiments proved hard to bring to the surface and to resolve and, in turn, may further compromise sustainable employability of healthcare workers and quality of care.

**Conclusions:**

In this study, we show how the relationship between the sustainable employability of healthcare workers and quality of care is set in motion by seemingly unrelated organisational practices. To benefit both healthcare workers and patients, leadership and healthcare teams are urged to prevent (undertows of) slumbering sentiments by recognising sentiments as important signals of dysfunctional circumstances and by effectively organising participatory practices that enable healthcare workers’ voice and input.

**STRENGTHS AND LIMITATIONS OF THIS STUDY**

- The use of different qualitative data collection methods, and a longer data-collection period, allowed for an in-depth exploration and representation of participants’ lived experiences.
- The guiding principles of reflexive thematic analysis allowed us to move beyond surface-level descriptions and instead construct underlying explanatory mechanisms that reflected deeper meanings and social dynamics.
- An interdisciplinary research team was indispensable throughout the analysis phase, as it challenged assumptions and enabled the exploration of alternative interpretations.
- Reflexive thematic analysis does not follow a linear chronological path, which means that analysis was slow, time-consuming and required ongoing critical reflection.

## Background

 The sustainable employability (SE) of healthcare workers (HCWs) is under pressure, as evident from widespread ill health, high sickness absence rates, increased turnover (intent) and recruitment challenges.^1^,^4^ SE refers to how individuals remain employed throughout their careers while simultaneously being able and enabled ‘to function as efficiently, effectively and healthily as possible within a given (un)employment context, now and in the future’.^5 6^ When the SE of HCWs is compromised, they are unlikely to function to the best of their ability.^7 8^ This impacts quality of care (QoC),^9^ for example, by leading to medical errors, missed care and unfavourable patient outcomes.^10 11^ It also threatens the availability and continuity of care, as HCWs with diminished SE are more likely to report sick or leave their jobs.^6 12^ If SE is strengthened, however, it improves patient safety culture, patient satisfaction and patient treatment adherence.^13^,^15^ QoC can also impact SE when viewed as an emotive experience.^8^ When HCWs perceive the quality of their care as low or experience an adverse event, they may experience moral injury, compassion fatigue, burnout or worse long-term consequences.^16^,^18^ When HCWs perceive QoC as high, their job satisfaction and productivity increase, whereas their intent to leave decreases.^19^ In sum, the SE of HCWs and QoC are mutually reinforcing and interconnected.^8 20 21^

Although associated, the relationship between HCWs’ SE and QoC is complex and situational, as it is constantly affected by societal, organisational and technological developments.^8^ Examples include ageing societies requiring more structural and chronic care,^22 23^ the introduction of new technologies,^24 25^ regulations affecting accountability requirements^26 27^ and policies to (de)centralise care.^28^ These changes prompt healthcare organisations to rethink and reorganise healthcare services and jobs to accommodate an increased number of patients with limited (human) resources, while simultaneously trying to sustain quality standards.^29^ The ways care and work are (re)organised and managed arguably affect SE and QoC, including their interplay.^8^ This is not necessarily negative, as organisational practices and jobs that are designed to take into account the health and well-being of employees may well contribute positively to quality healthcare provision.^30^ Especially when such endeavours acknowledge that sustaining quality care services requires an engaged and empowered workforce.^31^ Empirical work on the (re)organisation of care and work, however, shows that certain practices may unintentionally exacerbate situations.^7 32^ For example, the global COVID-19 outbreak prompted systems and organisations to radically change the provision of health services. Yet, in that process, many failed to adequately equip, protect and support HCWs to deal with larger caseloads, risk of infection and daily adversity.^7^ This ‘institutional and sociopolitical disinvestment in HCWs’ well-being’ not only harmed HCWs’ ability to provide good quality care, but also impaired their health and inclination to stay employed in healthcare.^7^ To avoid such outcomes, some scholars argue that healthcare should be organised, financed and managed in ways that take the SE of HCWs into account if striving to sustain QoC goals.^33^ In other words, QoC, HCWs’ SE and the organisation of care/work should be constantly considered in light of each other.^30^ Unfortunately, empirical research that considers all three facets at the same time is scarce, despite a need for more integrative research connecting organisational practices, HCWs’ SE and QoC in order to maintain and advance quality care delivery.^30^

To better understand the intertwined relationship between SE and QoC and decipher underlying mechanisms, it is necessary to look at organisational practices, such as how care and work are organised and managed.^8^ For example, research shows that if HCWs are enabled to voice patient safety or work-related concerns, they are more satisfied and committed to their organisation, have better well-being and, in turn, are more inclined to take ‘follow-up action’ for the benefit of both.^34^ On the other hand, more active involvement in addition to already intense job demands may take too much of a toll on HCWs who are already overwhelmed, possibly contributing to change fatigue.^35^ In this paper, we aim to further explore the relationship between SE and QoC by examining organisational practices in two hospital teams that work on improving specific clinical processes.

## Methodology

### Study design

This research presents data that were gathered in an action research project,^36^ which examined how accountability practices impact reflection and discussion on quality in healthcare teams. Six teams at three Dutch hospitals were shadowed between September 2021 and July 2022 in their efforts to improve specific clinical processes.^37^ The study reported here analyses data from two teams at one of those hospitals. As we aim to unravel underlying—likely invisible—mechanisms that relate SE and QoC, our research is guided by the principles of constructivism.^38^ This paradigm emphasises how a deeper meaning behind social phenomena is best understood through individuals’ own descriptions and constructions.^38^ Accordingly, this research uses qualitative research methods in line with the principles of reflexive thematic analysis (RTA) to collect and analyse these subjective experiences.^39 40^ RTA is an ‘artfully interpretative thematic analysis approach’ that aims to produce themes that each represent a recurring pattern of meaning across a dataset and which, in turn, may spark reflection or debate as to its significance or implications for ‘the wider social context’.^39^

### Setting

Research was conducted at a top clinical teaching hospital that had merged out of two local hospitals several years earlier. After the merger, its separate locations remained, which we refer to as location X and Y. The first team that participated in this research included scrub nurses from the operating room (OR), and the second team included nurses from the Acute Short Stay (ASS) unit of the emergency room (ER). The OR was operational at both locations, and the ER was located at location Y. Boxes1  2 provide more detail on the clinical processes.

Box 1 **Clinical process operating room (OR): preventing the retention of surgical items**

Surgical teams are responsible for preventing retained surgical items in patients undergoing surgery, such as sponges and instruments. At this hospital, scrub nurses do so by counting surgical items before surgery and by recounting right before incisions are closed to make sure that numbers align. However, the ways in which scrub nurses count differed between locations. Scrub nurses originally employed at location Y only count those surgical items that are required for a specific surgery. They usually count by themselves and by heart. Sponges and other smaller items that are used during surgery are typically counted out loud, so another scrub nurse can confirm. Scrub nurses originally from location X are used to counting according to a protocol that was developed following two incidents before the merger. The protocol stipulates that two pairs of eyes (either an extra scrub nurse or logistics employee) are to count the *entire* surgical kit before and after surgery with the help of counting lists. After the merger, both locations kept their operating rooms, but surgical teams would now rotate, meaning that scrub nurses originally from location X and Y would now work alongside each other. Management had received signals from scrub nurses originally from location X who were distressed about the counting practices at location Y, and as such considered it prudent for both locations to adopt the same counting practices. The head of department intended for scrub nurses to agree on uniform counting practices by themselves, rather than imposing a certain method. However, in practice, uniformity was tried to be realised by implementing the protocol developed at location X at both locations by team management. This met with a lot of resistance, especially as scrub nurses from location Y were perceived not to be taken seriously despite serious fundamental and practical objections.

Box 2 **Clinical process emergency room (ER): appropriate transfer to the acute short stay (ASS) unit**

After the merger, only the ER at location Y remained open. To accommodate a larger influx of patients with limited resources, in particular due to a shortage of ER nurses, the department created a separate unit at the ER called the ASS. This unit was meant for ER patients who were relatively stable, low-complex and expected to be able to go home (rather than be admitted), but had yet to await test results or (follow-up) consults with a specialist. It was reasoned that transfer of these patients from the high care unit to another unit would avoid ER beds being occupied unnecessarily long, and as such, could decrease overcrowding at the ER. The ASS was set up at the right side of the ER patient entrance, while the main unit of the ER (high care) and management’s offices were located at the left of the entrance, meaning that face-to-face interaction was sparse between units. Although there were criteria for physicians and ER-nurses to determine eligibility for transfer to the ASS, management questioned whether it was used appropriately; sometimes it appeared that patients were not transferred despite eligibility, other times patients were transferred but did not seem to be totally eligible. The ASS is staffed by vocationally trained ASS nurses, most of whom were recruited from departments within the hospital (eg, cardiology). During shifts, ASS nurses work in pairs while attending to a maximum of 14 patients.

### Patient and public involvement

Patients and/or the public were not involved in the design, conduct, reporting or dissemination plans of this research.

### Reflexivity

The research team comprised researchers with expertise in occupational health, healthcare regulation, healthcare governance and governance of professional work. We draw from a multitude of disciplines: organisational/occupational psychology, medicine, biomedical/health sciences and sociology. In addition, all have ample experience in qualitative research. This disciplinary diversity allowed us to notice and reflect on the relevance of HCWs’ SE within the quality improvement (QI) work of the examined departments and, as such, inspired this particular research study. Each team member brought a unique perspective based on their disciplinary background and individual professional roles. For instance, members with an academic background and/or work experience in healthcare regulation were able to offer valuable insights into the broader regulatory context in which HCWs operate. This perspective likely shaped our understanding of how external regulations and standards influence the QI practices within the departments studied. Likewise, expertise in occupational psychology and healthcare governance provided a lens to explore the potential importance of organisational culture and group dynamics for HCWs’ SE. Finally, a background in sociology brought attention to power dynamics and social structures within hospital teams. All of these different insights were crucial in producing an analytical ‘story’ of our data, as each researcher was able to notice different (potential) meanings behind codes, prompting further debate and reflection, and additional lenses for further theme development.

### Data generation

Data collection took place in two phases. The first phase, spanning several months, aimed to develop a comprehensive understanding of the clinical processes—how these were designed, organised and experienced—from multiple perspectives. Following the principle of crystallisation,^41^ which advocates using diverse data sources to establish a ‘complex, in-depth, but still thoroughly partial, understanding’, we combined semistructured interviews with undisguised non-participatory observations.^41^ Before data collection, we introduced our project to HCWs during staff meetings and group chats to ensure familiarity with our presence. Here, HCWs were also able to suggest potential interviewees and observation opportunities. Interviews focused on participants’ experiences and views of the clinical processes in terms of their intent, design, organisation and unfolding in practice, and were conducted with individuals directly involved or knowledgeable about them. A topic list can be found in online supplemental file 1. Participants—identified with the help of HCWs and quality and safety advisors—included those individuals that were likely to be ‘difficult’ to observe, referring to those less visible on the workfloor (eg, managers) or those being potentially influenced to behave or answer in a socially desirable manner (eg, interns). After obtaining written consent, ten interviews were conducted: four at the OR (one department manager, two supervisors, one intern scrub nurse), and six at the ER (one department manager, one medical manager (ie, physician with managerial responsibilities), one supervisor, two ER physicians and one ER nurse). Each interview lasted between 45 and 60 minutes, was audio recorded and transcribed. Observations focused on collaboration, social conventions, relationships and atmosphere in relation to the clinical processes. With verbal consent, we either shadowed individual HCWs in different places, or observed different individuals at specific sites. Ongoing consent was maintained by relocating or discontinuing observations if discomfort was felt or relayed. In total, 49 hours of observations were conducted: 29 hours at the OR and 20 hours at the ER. The duration of phase one was guided by the concept of information power,^42^ which estimates sample adequacy based on the quality or richness of collected data. Data collection ceased once all key stakeholder perspectives were covered, leaving us with a multilayered understanding of the clinical processes. Findings from the first phase were presented to staff and management for feedback and reflection, informing subsequent improvement interventions (eg, team coaching) within teams. The second phase involved observing staff and management meetings related to these interventions.

### Data analysis

We used a reflexive approach to thematic analysis (RTA) with the intent of uncovering both overt and latent meanings from participants’ perspectives, opinions and experiences.^43^ This qualitative approach to TA acknowledges how coding and theme development is a reflexive, subjective, flexible and iterative process.^43 44^ Accordingly, we do not report a linear stepwise approach or chronological ‘recipe’ for the themes we report on. Instead, themes were created gradually as a result of prolonged immersion and active engagement with the data. This means moving back and forth between phases of data familiarisation, code generation, theme construction, potential theme reviewing, theme naming and producing an account of themes.^44^ For an elaborate account on how we moved through these different phases, consult online supplemental file 2. Furthermore, we used an inductive approach to data analysis that was, however, coloured by our theoretical assumption that SE and QoC are interconnected and situational. RTA considers knowledge and meaning to be ‘situated and contextual’ and, as such, stresses that the subjectivity of researchers should be regarded as a ‘resource for knowledge production, (…) rather than a must-be-contained threat to credibility’.^45^ Nonetheless, analysis was a group effort, with each phase of analysis subject to frequent discussions and brainstorms with the entire research team.^46^ To further ensure quality analysis practices, we consulted and considered Braun and Clarke’s ‘ten common problems in published TA research’.^45^

## Analysis

For each of the three themes, we first describe specific organisational practices in both departments. Second, we show how these practices affected SE and QoC separately and in interaction. Third, we demonstrate how these effects contributed to slumbering negative sentiments among HWCs that, when left unaddressed, transformed into a brittle environment, which we call the ‘undertow’. The identified undertows were invisible but palpable, harboured different sentiments and may continue to constitute a suboptimal environment for sustaining HCWs’ employability *and* constructive QI work. We also show how these undertows were hard to address or resolve, as deeper sentiments underlying these undertows were generally cloaked, presented as surface level objections, or were not easily voiced by HCWs. For each theme, we illustrate our points with empirical data from either the OR or the ER. For a full overview of arguments for both departments, see table 1. Sentences or words in italics reflect the exact wordings of participants.

**Table 1:** Organisational practices that affect SE, QoC, their interaction and how negative sentiments develop into undertows

Theme	Organisational practice	Mechanism	SE	QoC	Interplay SE and QoC	Undertow
*Running on empty*	Organising care	Ongoing job strain that was not adequately or timely addressed	**ER:** impaired physical and mental health; turnover intent; affective rumination; unconstructive ventilating	**ER:** inability to deliver care according to (own) quality standards; undermined readiness to help other colleagues; lack of time to defuse irritations; higher likelihood to forget tasks/information	Routinely overburdened staff compromised staff’s ability/willingness to cooperate/coordinate care, affecting QoC. Delivering care below staff’s standards, in turn, compromised staff’s well-being	Undertow of discontent
			**OR:** high sickness absence rates; planning issues; short fuses/bad tempers	**OR**: lack of time to attend to QI; unable to follow protocol (and feeling unsafe); apprehension to voice (quality) concerns; forced to choose between different tasks/responsibilities		
*Us vs them*	Employing staff	Perceived distance between staff members/groups was not recognised or effectively countered	**ER:** unbelonging; feeling left out/forgotten; difficulty acquainting with colleagues; tit-for-tat dynamic	**ER:** difficult collaboration; unconstructive assumptions about the ‘other’	Not feeling at home or to not belong complicated collaboration among staff. Difficult collaboration, in turn, affected staff’s sense of belonging	Undertow of distrust
			**OR:** feeling out of place; unbelonging	**OR:** perceived complicated collaboration/coordination; unwillingness to concede in QI endeavours; decreased willingness to help ‘other’ colleagues		
*Missing the point*	Managing QI work	Fundamental concerns by staff members regarding quality or work-related issues were routinely disregarded	**ER:** feeling unheard, disregarded or unappreciated; tensions between colleagues/between staff and management	**ER:** apprehension to voice (quality) concerns; passiveness towards QI	Not being enabled or empowered to engage in QI work affects staff’s joy-at-work. Impaired job motivation and/or lower morale, in turn, affects staff’s willingness to raise QoC concerns	Undertow of inertia
			**OR:** dissatisfaction with leadership (spilled-over into other domains); reluctancy to engage in extrarole behaviour	**OR:** unresolved safety issues; staff feeling unsafe; uneasiness addressing safety perception; indefinite QI endeavour; disillusionment with QI		

ER, emergency room; OR, operating room; QI, quality improvement; QoC, quality of care; SE, sustainable employability.

### Running on empty

#### Organisational practice/change

The ways in which care was organised at the OR and ER after the merger contributed to prolonged heightened workload and pace and greatly overtaxed HCWs, which in turn impaired their SE and QoC. The ASS unit of the ER, for example, was created at location Y to accommodate for a greater influx of patients with fewer resources when the ER at location X closed as a result of the merger. When the High Care unit of the ER became (over)crowded, the incentive to use the ASS increased for ER staff. This, however, often meant that patients were transferred to the ASS in bulk rather than gradually, which significantly increased the workload of ASS nurses at peak moments. In addition, ASS nurses often felt that the requirements for transfer to the ASS—stable and low-complex patients—were being stretched in busy times by ER staff, increasing the workload per patient as well.

#### Effects on SE, QoC and their interplay

There were multiple implications of routinely overburdening staff. A few ASS nurses mentioned how their physical and mental health suffered under continuous demands, a state that was already fragile because of past and ongoing COVID-19 care. Some had even bordered on burn-outs. Furthermore, a high workload affected nurses’ ability to deliver care according to their own quality standards, which had led some to start looking for different jobs.

An ASS-nurse tells me that she often bursts out into tears; it is that crowded. She tells me about this situation where a patient died and that there was no time to sit with the family and to break the news to them. (fieldnotes ASS)

In addition, ending one’s shift when it was still very busy made many ASS nurses uneasy, worrying if they left everything in good order. Moreover, ongoing job strain resulted in “*unconstructive ventilating*”, “*snappiness*” and “*short fuses*” among staff, particularly between ER nurses/physicians and ASS nurses. This undermined staff members’ willingness to help each other, and this may have compromised patient care since most care at the ER requires different HCWs to collaborate and coordinate care. Because of the high workload, there was not much time to defuse these irritations either. Furthermore, staff explained that there was a greater likelihood of forgetting tasks or informing patients and/or caregivers. The transfer of patients during busy moments appeared especially “*error prone*”. In sum, when job demands were routinely overburdening staff, their willingness and ability to cooperate and coordinate care became compromised. In that process, QoC may become compromised as well. In turn, when staff perceived that they were delivering care that was below (their own) standard, it affected their well-being.

#### Undertow of discontent

Over time, continuous dissatisfaction with structurally high workload and pace without adequate relief transformed into an invisible but palpable ‘undertow of discontent’. At the ASS, several ASS nurses had relayed the consequences of prolonged heightened workload to management on several occasions. They had advocated for an extra (third) ASS-nurse during structurally busy afternoons. Nurses spoke of a “*battle*” to convince management of this perceived necessity. It apparently took a nurse to “*cry out in desperation*” before management intervened. However, rather than an additional nurse, they received a medical assistant, which some perceived to be an impractical solution, as they could not help with direct patient care. When this undertow of discontent was eventually acknowledged, ASS nurses had already become increasingly dissatisfied, but as that sentiment had been met with neglect in the past, HCWs had more or less stopped sharing this sentiment. Instead, ASS nurses mentioned how a “*culture of complaining*” had emerged*,* where they ventilated and complained among each other. Most of them did not consider that dynamic productive or constructive for QI or team dynamics.

Nadia [ASS-nurse] thinks that the “*culture of complaining*” or the discontentment will keep being reinforced because new staff will be dragged along in that atmosphere. (fieldnotes ASS)

### Us versus them

#### Organisational practice/change

The ways in which staff were employed at the OR and ER after the merger contributed to prolonged perceived distance—either physical or cultural—between HCWs from the same department, which, in turn, cemented ‘local’ identities and created an ‘us’ versus ‘them’ dynamic that affected SE and QoC. For example, the head of the OR department decided to let staff rotate between the two locations of the merged hospital to facilitate interaction, the latter considered a prerequisite for creating uniform counting practices. However, rotating staff reinforced and strengthened local cultures rather than fusing them. This may be attributed to the process of finalising the merger, when separate ORs from both locations needed to streamline their services. This process was characterised by many discussions between locations, even at seemingly trivial levels, such as which brand of sponges to use henceforth.

During lunch, I ask James [logistics employee] why after all this time people still speak of ‘location X’ and ‘location Y’. James explains that the X/Y identity lingered because the merger was *“messy”*. Everyone still speaks in terms such as ‘we’, ‘they’, and ‘at us’. (fieldnotes ASS)

Moreover, the protocol for preventing the retention of surgical items, which had been developed at location X prior to the merger, was perceived by scrub nurses from location Y to have been *“copy-pasted”* to their context without adequately involving them or listening to their fundamental concerns. When scrub nurses had to work at a location other than their ‘original’ one, they perceived a very strong cultural norm and little to no room for counting instruments in their preferred way. The ways in which surgical items were counted became symbols of local identities and of belonging somewhere. As a result, both locations adhered religiously to their preferred counting method, with little incentive to create uniform counting methods.

#### Effects on SE, QoC and their interplay

In terms of QoC, physical and cultural distance felt between HCWs discouraged collaboration, coordination and improvement of care, as well as extrarole behaviour (eg, helping ‘the other(s)’), which contributed to tensions. In terms of SE, staff did not feel at home or to belong when working at the ‘other’ location. Feeling out of place, in turn, further complicated collaboration, which, in turn, impeded staff feeling at home at other locations/units.

Today I have joined scrub nurses Antoinette and Mary at location Y, who will assist a surgery that I get to attend. (…). The surgeon explains that if he works at location X, he feels *“like a fish out of water”*. I ask Antoinette about working at location X vs location Y. (…) She thought the atmosphere was different, more docile, also with surgeons. *“Here, we have no problem talking back to a grumbling surgeon”*. Working there did not suit her, and she experienced much friction. She did not feel at home. (fieldnotes OR)

#### Undertow of distrust

Over time, in the absence of an adequate and timely response to bridge differences, this dynamic of strong local identities resulted in an invisible but palpable ‘undertow of distrust’. Once established, it proved difficult to address this undertow and to let the accompanying sentiment surface. At the OR, one of the Q&S advisors suggested an intervention to reconcile the two groups (pro- and con-counting protocol) by organising ‘deep democracy’ sessions. Such sessions are meant to address conflicts or undercurrents in groups through respectful listening and dialogue. Two of these sessions were organised for a mix of scrub nurses from both locations. Team managers were also present. For scrub nurses, these sessions were the first formal time since the start of the QI project where they were given the opportunity to discuss the matter among themselves directly. Despite the intent of the session, it was difficult for the group to listen carefully to others and to not interject. There was a lot of boiled-up frustration and scepticism as to what the sessions could resolve after all those years. Moreover, arriving at the deeper layers, or the undertow, was difficult. HCWs seemed more comfortable expressing their discontent with practicalities—more surface level issues, such as misplaced counting checklists—than discussing interpersonal relations. After the 1.5-hour mark in one session, many topics were discussed, yet without any explicit resolution between ‘both camps’, leaving most of the scrub nurses even more disillusioned.

Mona (Q&S advisor) concludes that the conversations do not seem to be finished at all. A scrub nurse replies: *“This conversation has been going on for 3 years already”*. Mona: “*How do you want to return to what has been discussed today?”* Someone else replies sarcastically: *“every month a work outing?”* Some scrub nurses start laughing, others do not seem to find it funny. Someone walks out of the room: *“we won’t ever resolve this”*. (fieldnotes OR)

### Missing the point

#### Organisational practice/change

The ways in which QI work was organised and staff were managed throughout contributed to staff members feeling routinely disregarded when voicing fundamental quality or work-related concerns, which affected SE and QoC. At the ASS, nurses were frustrated by the lack of opportunity to share their side of the story on the functioning of transfer to the ASS. For example, they mentioned how their team meetings were mostly structured around the team leader’s announcements, with limited space to raise issues themselves. In addition, ASS nurses stressed that they were often unable to attend the ‘end-of-shift’ evaluations with the other ER-units (in a timely manner) because those evaluations coincided with their own shift changes. This limited their understanding of the other unit’s perspectives on ASS transfer issues. Moreover, owing to a lack of continuous supervision as a result of numerous manager turnovers in the past 5 years, many signals from ASS nurses regarding QoC had not been taken up properly by management to the dismay of staff. As a result, some ASS nurses questioned the added value of reporting quality issues:

“*(…) I hear this thing again about quality. Sure, I do acknowledge the importance of feedback from the floor, but there is never follow-up. Am I only meant to merely think about quality then?*” (fieldnotes ASS)

ASS nurses also complained about the ‘missing’ face of management. The team leader had her office at the left side of the ER entrance, whereas ASS nurses worked at the right side, which limited the opportunity for ASS nurses to report QoC issues directly:

ASS-nurse*: “Even though you [team leader] do visit sometimes, you will hear so much more when you sit with us. Then you truly experience what happens on the floor. I get that your office is somewhere else, but perhaps you would be able to sit in our break room [also the workspace of ASS nurses] when it is busy to truly understand the ins and outs here.”* (…) (fieldnotes ASS)

#### Effects on SE, QoC and their interplay

To regularly feel unseen or unheard when voicing QoC issues, fused tensions between staff—as they were insufficiently able to share their perspectives and create mutual understanding—and between staff and management. In addition, it made staff feel disillusioned with and passive towards QI and made staff reluctant to engage in extrarole behaviour, such as lending a hand towards their colleagues. However, as staff were passionate about their work and improving QoC, not being enabled or empowered to effectively engage in QI work was a substantial disappointment for HCWs’ joy-at-work. In turn, this lower morale was not helpful in motivating staff to continue raising concerns with all the obstacles in place, to the detriment of QoC.

#### Undertow of inertia

Over time, as HCWs were neither sufficiently offered the opportunity to voice concerns nor were taken seriously when they expressed their worries, an ‘undertow of inertia’ materialised, with HCWs doubting the relevance of speaking up and sometimes avoiding it altogether.

Nadia [ASS-nurse] acknowledges that there is *“passivity”* at the ASS. Maria [ASS-nurse] explains that this attitude stems from issues in the past that have not been addressed properly within the team by management. *“I am sure that half of us think that way”*. Kate [nurse quality officer] explains that people have experienced some things (…), which *“has left their marks”*. (fieldnotes ASS)

At the ASS, the team leader addressed this undertow through team coaching, which was facilitated by a trainer from the in-house academy during one of the regular ASS-team meetings. Once again, however, it proved difficult to surface that underlying sentiment. This may be because the ASS team had not been directly informed of the intent of the intervention—which was to overcome QI ‘passiveness’—but was instead told that it was intended to improve internal group dynamics. Unsurprisingly, when the ASS-team was given the floor to unload, vent or clear the air, nurses remained as good as silent and quickly returned to surface-level topics:

Lela [team leader] starts with an introduction: *“(…) a lot has happened since we started with the ASS, this presentation may serve as something to look back on, are there still issues bothering you? Do you want to share anything? So there will be closure, and space to move on, to have good team dynamics once more.”* (…) the group does not seem to have to discuss much: *“There is nothing bothering me”*. After a short silence, the discussion steers back to the process of transferring patients to the ASS. (fieldnotes ASS)

## Discussion

This research aimed to better understand the underlying mechanisms that explain the intertwined relationship between the SE of HCWs and QoC. By exploring organisational practices in a post-merged hospital, this study identified three mechanisms as a result of different organisational practices that affected SE and QoC separately and set in motion their interconnectedness: routinely overburdened staff due to the way care was organised, prolonged perceived distance between staff as a result of how staff were employed, and regular disregard of raised concerns by staff due to the way quality improvement (QI) work was organised and managed. Over time, as these mechanisms remained unaddressed, undertows of slumbering sentiments—of discontent, distrust and inertia—emerged, which proved very difficult to bring to the surface and resolve and, in turn, may further compromise SE and QoC.

Our main contribution is showing that SE and QoC are interlinked phenomena and that their relationship can be set in motion by seemingly unrelated organisational practices. Moreover, the mechanisms and undertows identified show *how* both interconnect. Following our findings, our main message is that slumbering sentiments, including the ways these manifest, should not be labelled as dysfunctional behaviour, but rather be viewed as coping mechanisms for HCWs to deal with, or persevere despite, dysfunctional circumstances.^47 48^ Accordingly, these sentiments should be regarded as signals that provide context information for both HCWs’ SE and QoC, which require genuine attention and further scrutiny, not ignorance or suppression.

This study’s evidence builds on other research streams. First, the identified mechanisms, the undertows and their effects on HCWs’ SE and QoC align with job demands-resources (JD-R) theory. In line with its reasoning, this study confirms that persistent high job demands deplete HCWs’ energy and eventually lead to ill health and impaired functioning at work, while a lack of (relational) job resources depletes HCWs’ motivation to achieve work-related goals, such as coordinating care or continuing to raise QoC concerns. ^49^ Moreover, when fundamental job resources (eg, managerial support, strong social ties among colleagues) are perceived absent, they can also no longer act to buffer the impact of high job demands, ^50^ even though high job resources were found to be even more functional in work settings where job demands are structurally high, ^51^ as is the case in ER and OR environments. As a result, employees who face high job demands combined with low job resources report ‘the highest levels of exhaustion and cynicism’, descriptions that are reminiscent of the slumbering sentiments described in this study^52^. Similarly, other studies have stressed how healthcare environments tend to misalign with essential needs of HCWs,^53^ making them feel unheard and unvalued, in turn leading them to experience difficulty in caring for their patients and reducing employees’ satisfaction towards their jobs or employers.^54^

JD-R theory also outlines how job strain as a result of high job demands often leads employees’ to engage in maladaptive self-regulation, such as coping inflexibility or self-undermining (eg, interpersonal conflicts), which further increases job demands and, in turn, job strain. ^55^ This study shows how coping mechanisms may not always be traced back to the individual, but rather present as collective phenomena in teams, departments or even organisations,^56^ where (new) team members may quickly align with the prevailing sentiment through non-conscious emotional contagion or more conscious attunement.^57^

Second, one explanation for why the different sentiments in groups were not timely or adequately addressed can be explained by literature on merged healthcare organisations. From their case studies, Fulop *et al*^58^ conclude that organisational culture plays a crucial yet underplayed role throughout mergers. Organisational groups tend to be attached to their established working patterns, and if one group perceives itself to be ‘taken-over’ by the other, for example, when certain practices are being imposed on them, it can cause resentment that may linger for years. In addition, these negative sentiments are often not observed properly due to delays in appointing sufficient middle managers during merger periods, which creates a leadership vacuum in which front-line staff are unable to voice their discontent and concern.^58^ However, mergers are not only framed as disruptive events, but may also serve as a time and place for reflection, where common, often rooted, care practices are reflected and potentially improved on.^59^ In addition, one study found that being heard, valued and supported throughout important transitions (a pandemic, life events or organisational changes such as mergers) sustained or improved the well-being of HCWs.^60^

### Recommendations for future research

Future research may supplement this study by exploring how different organisational practices relate to SE and QoC. In addition, the undertows that are described in this research may be explored further in different settings to determine if these are common phenomena, whether they harbour similar or different sentiments, and to what extent or how they affect HCWs’ SE and QoC. Although this study reported a downward spiral for SE and QoC as a result of organisational practices, future research is encouraged to explore how certain practices contribute positively to SE and QoC.

### Evaluating our approach

We acknowledge that the context of this research may be specific to the account that is eventually produced here.^40^ The merging of this hospital, although finalised on paper, clearly continued to leave a mark on participants’ daily work and may have exacerbated the negative sentiments as described. In addition, this research took place during the COVID-19 pandemic, a highly stressful, taxing and uncertain time for both managers and HCWs. This may have equally rendered HCWs’ susceptible to negative sentiments. Throughout the conduct of our research, we did encounter some challenges. Although a focal point of our research, QI was not a priority in practice, and accordingly, the second phase of our research on improving QI practices in teams arguably did not materialise as expected, which limited our ability to evaluate their aftermath. In addition, again attributed to the commotion of COVID-19, (team) managers were—understandably—not always very responsive to our call for further information or organising meetings to discuss research results and follow-up, which limited our ability to collect data at certain moments. Having said that, we very much like to advocate for reflexive thematic analysis. Although it may prolong the analysis phase significantly, in hindsight, it did enable us to ‘see’ and interpret different potential meanings from the data, contributing to its richness. Interdisciplinary research teams are arguably indispensable for such reflexive processes.

### Recommendations for practice

Although the call for well-being and quality metrics is an undisputed important one, it is generally a call for more measurable data, such as employee surveys, checklists, or quality indicators.^61^ Following our findings, we recommend that such metrics should also incorporate qualitative information (eg, soft signals or soft intelligence) to grasp why and how SE and QoC interact in certain settings.^62 63^ This, however, requires a different form of ‘data collection’. Specifically, we advocate for more inclusive, involved and empathic leadership practices, where managers and supervisors are aware and attentive to HCWs’ needs and concerns, especially during and after periods of extensive organisational change such as mergers or pandemics. Specifically, (middle) managers should make a conscious effort to empower HCWs’ participation in decision-making processes and enable regular reflection and dialogue in healthcare teams.^64 65^ This also implies that healthcare leaders, especially frontline supervisors, should be enabled to engage more frequently and directly with frontline HCWs to truly grasp (ie, sense and feel) the meaning behind these signals.^66 67^ Currently, this vision is still in stark contrast to the prevailing leadership paradigm in many healthcare settings, characterised by top-down, controlling practices and measurable performance indicators.^65^ In addition, we wish to emphasise that leadership is not solely reserved for leaders but should be a collective and dispersed practice,^65^ meaning that also HCWs—both individually and as a team—should equally contribute to fostering the abovementioned practices. We believe that these recommendations can prevent healthcare teams from being caught in an undertow, instead strengthening the foundation for better HCWs’ SE and QoC, and ultimately improve the care experience of both care providers and their patients.

## Conclusions

In this study, we have shown how SE of HCWs and QoC are inextricably interconnected, and how their relationship is set in motion by seemingly unrelated organisational practices. To benefit both HCWs and patients, leadership and healthcare teams are urged to prevent (undertows of) slumbering sentiments by recognising sentiments as important signals of dysfunctional circumstances and by effectively organising participatory practices that enable HCWs’ voice and input.

## Supplementary material

10.1136/bmjopen-2025-108470 online supplemental file 1

10.1136/bmjopen-2025-108470 online supplemental file 2

## Data Availability

Data are available upon reasonable request.
