# Supplementary material for: Caught in the undertow: a qualitative study exploring the relationship between the sustainable employability of healthcare workers and quality of care
Source: BMJ Open. 2025 Dec 14;15(12):e108470. doi: 10.1136/bmjopen-2025-108470 (PMC12706189; doi:10.1136/bmjopen-2025-108470)
Supplement: online supplemental file 1 [file bmjopen-15-12-s001.docx]

**Online supplemental file 1: interview topic list**

**Introduction**

**Function and roles**

- Can you tell us something about your position?
  - Professional background?
  - How long have you worked in the team/on the clinical process?

**Clinical process**

- What does the process look like?
  - What is being done by whom?
  - At what times?
- What are your experiences with the clinical process?
  - What is going well?
  - Are you running into something? Challenges in the process?
- To what extend do you feel your vision or perspective regarding the process differs from others?
  - Different perspectives/interests?

**Experiences with mutual feedback and reflection**

- How does the team learn/reflect/give feedback on what is going well? And on what goes less well?
  - With whom? (team, who are they?)
  - At what times; formal or informal?
  - Where?
- What space or time is there to address each other about what is going well/not so well?
  - Which connections/facilitate reflection/feedback
  - What is the influence of accountability?
- How do you view the importance of addressing each other?
  - Why is that important?

**Experience with hierarchical accountability for the clinical process**

- What are you accountable for with regard to the process?
  - To whom do you account?
  - When are you accountable?
- What is your experience with hierarchical accountability with regard to this process?
  - What is the influence of accountability on the process?
- What could be done better? How can more be in line with practice?

**Closing remarks**

- Do you have any questions/additions/comments?
