# Supplementary material for: Caught in the undertow: a qualitative study exploring the relationship between the sustainable employability of healthcare workers and quality of care
Source: BMJ Open. 2025 Dec 14;15(12):e108470. doi: 10.1136/bmjopen-2025-108470 (PMC12706189; doi:10.1136/bmjopen-2025-108470)
Supplement: online supplemental file 2 [file bmjopen-15-12-s002.docx]

| Supplemental file 2: Detailed account of analysis through different phases |
| --- |
| Phase 1: familiarisation |
| Analysis began at the end of 2024, requiring us to (re)familiarise ourselves with data collected in 2021-2022. Also, only I (the first author) had been directly involved in data collection, with the third and fourth authors – as project managers – knowing the setting through my accounts. Accordingly, it was necessary to collectively revisit the clinical processes, health care teams and the hospital context to develop a shared understanding of the setting. I (the first author) did so by rereading all fieldnotes and transcripts, trying to reconstruct the teams’ histories with the clinical processes by highlighting crucial and vivid passages that illustrated significant moments in (chronological) time. These were shared during biweekly team meetings. |
| Phase 2: coding |
| I (first author) systematically coded each interview and fieldnote in ATLAS.ti (version 25), starting with the OR and subsequently the ER/ASS. Given this study’s exploratory nature, coding was initially inductive and semantic, staying close to participants’ explicit language and meanings. To manage the extensive list of codes, I merged overlapping codes after each transcript or fieldnote, keeping my list of codes organized and ‘clean’, as I had learnt (the hard way) from earlier qualitative analyses. Codes pertaining uniquely to the OR and ER could still be retained with the help of ATLAS.ti, so none of the original (local) meanings would be lost. I frequently revisited earlier coding material to refine or recode excerpts as new insights emerged. This detailed and thorough coding process enabled me to carefully reconstruct how the clinical processes had unfolded in practice from participants’ perspectives. In our biweekly meetings, I would share codes and excerpts that had been created (so far) to reflect on the role of SE in the health care teams’ enactment or improvement of clinical process, which served as input for discussion and brainstorming. |
| Phase 3: initial theme generation |
| In this phase, I explored patterns of meaning among across both the OR and ER/ASS, by comparing similar codes and discussing potential overlaps with the research team. Here, we discerned four preliminary themes which were still loosely coupled to our research question and required further scrutiny/debate: 1) moral distress among HCWs; 2) (similar) coping mechanisms; 3) Lack of time to address unpleasant situations, and 4) a strained relationship between staff and management. |
| Phase 4: reviewing and developing themes |
| As our study sought to explore mechanisms linking HCWs’ SE to QoC (and vice versa), I began by reflecting on how themes could exactly relate to our research question, writing my initial interpretations in draft papers. These drafts were shared with the research team, prompting collective discussions in which we critically reflected on the extent to which themes were able to tell a story that would provide an answer to the research question. This collaborative phase was iterative and arguably the most time-intensive, involving multiple cycles of brainstorming, rereading, and reformulating themes. Through this process, we slowly but gradually finetuned our final themes until we had a convincing story to tell about how SE and QoC continuously seemed to interact in practice. This is when we gradually recognized and constructed the idea of underlying slumbering sentiments that emerged as a result of specific organizational practices, which continued to affect both SE and QoC (continuously). |
| Phase 5: refining, defining, and naming themes |
| We experimented with various theme labels to capture what exactly was happening in the teams as a result of these organizational practices. Ultimately, we aimed to present the themes as if these represented answers from health care teams if we would have asked them ‘how is it going? – to which they might have replied: ‘(I am) running on empty’, ‘(it is) us versus them’, and ‘(they [managers] are) missing the point’. |
| Phase 6: producing the report |
| Although presented as the final phase of analysis, this stage unfolded alongside earlier analytic phases. Throughout the process, we continuously revisited our codes and themes and outlined these in several drafts that served as input for discussion: could we convincingly connect the analytic “puzzle pieces” within each team and more importantly convey a coherent story on paper that would address our research question. As for the final report, we decided that given the distinct contexts (despite patterns of shared meaning) we would foreground one team within each theme. We also decided that it would be coherent if each theme encompassed the same elements in the narrative: 1) the organizational practice in question; 2) its effects on SE, QoC and their interplay, and 3) the underlying sentiments that emerged in teams and how teams sought to address these. |
