# Supplementary material for: Caught in the undertow: a qualitative study exploring the relationship between the sustainable employability of healthcare workers and quality of care
Source: BMJ Open. 2025 Dec 14;15(12):e108470. doi: 10.1136/bmjopen-2025-108470 (PMC12706189; doi:10.1136/bmjopen-2025-108470)
Supplement: Supplementary file 3 [file bmjopen-2025-108470.reviewer_comments.pdf]

PEER REVIEW HISTORY

BMJ Open publishes all reviews undertaken for accepted manuscripts. Reviewers are asked to complete a checklist review form and are provided with free text boxes to elaborate on their assessment. These free text comments are reproduced below.

ARTICLE DETAILS

Title (Provisional)

Caught in the undertow: a qualitative study exploring the relationship between the sustainable employability of health care workers and quality of care

Authors

van de Voort, Iris; Leistikow, Ian; Bal, Roland; Weenink, Jan-Willem

---

VERSION 1 - REVIEW

**Reviewer**	**1**
**Name**	**Juvonen-Posti, Pirjo**
**Affiliation**	**Finnish Institute of Occupational Health**
**Date**	**03-Sep-2025**
**COI**	**None**

This article presents a truly interesting and important theme. Although observation in the described jobs and workplaces as a form of data collection is laborious and both ethically and practically challenging, this study has yielded rich data. I am particularly pleased with the way the researchers have succeeded in summarizing their results.

I propose to make some minor clarifications and additions:

Methods (4.): Reflexive thematic analysis is a good methodological choice for analyzing this data. However, there is more recent methodological literature on thematic analysis than what is referenced in the article, for example, Braun, V. & Clarke, V. (2022) Thematic Analysis: A Practical Guide, Sage Publications, which the authors should consider reviewing.

The authors describe well the non-linearity and iterativeness of the reflexive analysis process at a very general level. I would like the authors to concretize with a few sentences what iterativeness meant in this analysis process and what possible themes were identified and (briefly) how they were developed during the analysis process.

Research ethics (in practise)(5.)

The authors describe well that the study underwent ethical review and that the interviews were based on consent. However, in workplace observations, there are stages of data collection where obtaining consent can be practically challenging. Therefore, I hope the

authors clarify how research ethics principles were implemented when data was collected during staff meetings. Although data collection did not target patients, observations were made in connection with patient work (involving individuals in vulnerable situations): how were patients informed about the presence of researchers?

References (8.) and Limitations (12.)

The authors didn't refer to the latest references:

Braun, V. & Clarke, V. (2022) Thematic Analysis: A Practical Guide, Sage Publications, which the authors should consider reviewing.

Braun, V., Clarke, V., Hayfield, N., Davey, L., Jenkinson, E. (2022). Doing Reflexive Thematic Analysis. In: Bager-Charleson, S., McBeath, A. (eds) Supporting Research in Counselling and Psychotherapy . Palgrave Macmillan, Cham. https://doi.org/10.1007/978-3-031-13942-0_2

Braun V, Clarke V. Supporting best practice in reflexive thematic analysis reporting in Palliative Medicine: A review of published research and introduction to the Reflexive Thematic Analysis Reporting Guidelines (RTARG). Palliative Medicine. 2024;38(6):608-616. doi:10.1177/02692163241234800

The authors describe the limitations of their study as the context and setting. This article has commendably and concisely described both the setting and context. I am unsure if the researchers in the "Limitations" section are referring to the generalizability requirement arising from the tradition of quantitative research, but if so, I believe they are incorrectly doing so in this article. The "generalizability" of qualitative research results stems from different paradigms. The discussion of the study's limitations should address the possible challenges of conducting organizational ethnography in these workplaces (see also the issues raised in the Ethics section) and, for example, the challenges of implementing reflexive thematic analysis (if any were encountered).

Reflexivity in reflexive thematic analysis:

I would like the authors to delve a bit deeper into the various ways reflexivity was manifested in their implementation of reflexive thematic analysis. The multidisciplinary nature of the research team is a good starting point: please also indicate which disciplines the team represented.

**Reviewer**	**2**
**Name**	**Lombarts, Kiki**
**Affiliation**	**Amsterdam University Medical Centres, Medical Psychology**
**Date**	**22-Sep-2025**

COI **None**

Thank you for the opportunity to review this study. I enjoyed reading this well-written paper. It offers a deep dive in team dynamics and the effects on SE and QoC.

That SE and QoC are related is not new and indeed well documented. The novelty and main value of this important study is the exploration of how SE and QoC may be impacting each other as well as how the organizational practices and (resulting) undertows as reported by the authors may affect both QoC and SE, and their interrelatedness. The qualitative approach and reporting offers concrete, tangible examples, and makes it easy to understand (how the authors came to) the results.

General comments:

Methods

The methodology raises some questions, and requires more words to understand and trust the robustness of the methods chosen, including underlying docs (i.e. observation guides), reflexivity and ethical approval for the study (in particular since the researchers observed at departments (OR and ER) where (many?) patients and HCW's were present). See some detailed questions below.

Discussion

The authors could be more elaborate in the discussing their findings. Most of the discussion is dedicated to practice recommendations, and, although relevant, (first) critically reflecting on their own findings on a more conceptual level is a missed step.

For ex. the results in terms of the undertows, raise questions for reflection. If they could exist independent of org practice/change, what does that to the undertows in relation to SE and QoC? In other words, could the undertows revealed emerge even when organizational change is not happening?

The reflection should include the specific context in which the study was conducted: two departments in a post-merge hospital. Hospital merges may offer specific or additional challenges going beyond the labelling as just 'an organizational practice/ change'. There is much literature on (hospital) merges, does this study align with that lit? This may be particularly important when thinking about recommendations for practice. These may need to be more related to successful mergers than leadership or anything else. (see for ex the syst review on barriers and facilitators to successful hospital mergers, by the Health Review Board)

In general, this paper would improve by positioning the findings of this study in the broader literature. The authors could try better guide the reader understanding their findings in relation to i.e. theories on organizational cultures, team effectiveness and/or occupational health psychology.

Detailed suggestions:

Abstract:

Conclusion . I'd suggest to report the main result in the first sentence, and not to repeat the question.

Introd

Line 121-123: Re the quote: pls explicate who said this and in what context. The fact that it is quoted raises questions. Alternatively rephrase and just keep the reference.

Line 124: The sentence: Empirical …..situations, needs clarification.

Line 126. PLs clarify what is meant by the HH paradigm. Offer the 3 pillars mentioned the explanation, or is that separate from the paradigm?

Main question: given the 3 pillars and aim of the study, I expect you to look at c.q. interview people about the organizational aspects of work or (line 153) organisational mechanisms in relation to the 2 pillars of SE and QoC. What is it exactly you looked for, or at, for that matter?

Line 153 suggests a very open perspective.

Line 155. Sentence seems incomplete.

Line 169: Data collection:

General: Was ethical assessment obtained or waived? Pls report the evidence of at least one of them.

Line 175: was an interview guide used? Pls provide. Was an observational guide used? Pls provide.

How were the fieldnotes analysed in this process? Together with, apart from the interview data? How did you combine or integrate them?

Reflexivity:

Deserves more words. What does each person's experience and specific role (i.e. the Inspectorate), mean for the perspective they brought to the table, and how did this (potentially) impact the discussions, or even the coding?

Results

Line 217:

I understand correctly you defined three themes based on the analyses.

Line 231-240: Various shifts are described here, not only organization ones.

I assume that when the ER was closed at location X, ER personnel from this location was transferred to location Y, along with the patients. Correct?

Was it 'just' an organizational, logistic change, or were there also budget or personnel reductions involved? I am trying to understand whether the overburdening of personnel had other potential causes than the reorganization of practice.

L 255: are these italic words quotes or codes or? Italics are used throughout the results section, so I suggest the authors clarify in the first paragraph of the Results section how they should be read and understood.

L 257: please explain how 'readiness' and 'coordination'?

L 261-264: Given the phrasing it is not clear whether QoC was indeed effected by the overburdening of HCW – as expressed by them -, or that this is the authors' interpretation (QoC may become compromised).

Discussion:

(See general remarks above)

L 445: employer or employee satisfaction?

L 447: The examples of the reverse/positive relationship was not explicitly reported in the results. Was it explored at all? If so, consider how to include it in the reporting, in order to claim the statement made in this sentence. We can not infer that the absence of a problem self-evidently means the presence of a success.

L 468-478: This is an important paragraph, it interprets and elaborates on the findings, and is therefore more than a rec for practice. I'd suggest to move it up in the discussion section. The practice rec more appropriately can start at L 478 …Specifically….

A conclusion is missing. Pls add.

VERSION 1 - AUTHOR RESPONSE

Comments reviewers	Response and revision co-authors
**Reviewer 1**	
1. Methods (4.): Reflexive thematic analysis is a good methodological choice for analyzing this data. However, there is more recent methodological literature on thematic analysis than what is referenced in the article, for example, Braun, V. & Clarke, V. (2022) Thematic Analysis: A Practical Guide, Sage Publications, which the authors should consider reviewing.	We sincerely thank you for referring us to more recent work by the same authors. Especially the reporting guidelines have helped us with avoiding 'positivism creep'.

2. The authors describe well the non-linearity and iterativeness of the reflexive analysis process at a very general level. I would like the authors to concretize with a few sentences what iterativeness meant in this analysis process and what possible themes were identified and (briefly) how they were developed during the analysis process.	We have written down a more elaborate account of the analysis process. In the text we refer to online supplemental file 2.
3. Research ethics (in practise)(5.)<br>The authors describe well that the study underwent ethical review and that the interviews were based on consent. However, in workplace observations, there are stages of data collection where obtaining consent can be practically challenging. Therefore, I hope the authors clarify how research ethics principles were implemented when data was collected during staff meetings. Although data collection did not target patients, observations were made in connection with patient work (involving individuals in vulnerable situations): how were patients informed about the presence of researchers?	We agree that consent should not be a checkbox exercise and should rather be established throughout the conduct of data collection (i.e., ongoing/continuous). Staff meetings were not so much a challenge, as prior to data collection, we were introduced to respective health care teams in one of their staff meetings where there was ample room for their questions and concerns. We even encouraged their expertise as to whom to talk to, or when/where to observe in order to grasp the clinical processes adequately and to also establish rapport. However, we did encounter some challenges in establishing (ongoing) consent in observations of health care workers' daily work (particularly at the OR when counting surgical instruments). We describe several measures that we took during observations to ensure (ongoing) consent.<br><br>In terms of patient consent, it is true that we did encounter some patients, although these were not as frequent as one may expect (and if occurring quite swift due to the high work pace). If we encountered patients, health care workers that we followed were quick to inform patients that there would be a researcher in the room that would observe the

	health care team, and that the researcher had no access nor interest in any (identifiable) patient or medical information. Patients had no objections to this. We have added this information in the method section under 'ethics approval'.
4. References (8.) and Limitations (12.) The authors didn't refer to the latest references:<br>Braun, V. & Clarke, V. (2022) Thematic Analysis: A Practical Guide, Sage Publications, which the authors should consider reviewing.<br><br>Braun, V., Clarke, V., Hayfield, N., Davey, L., Jenkinson, E. (2022). Doing Reflexive Thematic Analysis. In: Bager-Charleson, S., McBeath, A. (eds) Supporting Research in Counselling and Psychotherapy . Palgrave Macmillan, Cham. https://doi.org/10.1007/978-3-031-13942-0_2<br><br>Braun V, Clarke V. Supporting best practice in reflexive thematic analysis reporting in Palliative Medicine: A review of published research and introduction to the Reflexive Thematic Analysis Reporting Guidelines (RTARG). Palliative Medicine. 2024;38(6):608-616. doi:10.1177/02692163241234800	Again, many thanks for these references. We have consulted the latter two and referred to them in the manuscript.
5. The authors describe the limitations of their study as the context and setting. This article has commendably and concisely described both the setting and context. I am unsure if the researchers in the "Limitations" section are referring to the generalizability requirement arising from the tradition of quantitative research, but if so, I believe they are incorrectly doing so in this article. The "generalizability" of qualitative research results stems from different paradigms. The discussion of the study's limitations should address the possible challenges of conducting organizational ethnography in these workplaces (see also the issues raised in	Thank you for notifying us of one of those instances where "undeliberate, unknowing entanglements with postpostivism" (Braun & Clarke, 2024, p. 613) seemed to have creeped in our manuscript (the 'positivism creep' is real). We have rewritten the limitations section (and rephrased this section 'as evaluating our approach') in accordance with the recommendations by Braun & Clarke (2024).

the Ethics section) and, for example, the challenges of implementing reflexive thematic analysis (if any were encountered).	
6. Reflexivity in reflexive thematic analysis: I would like the authors to delve a bit deeper into the various ways reflexivity was manifested in their implementation of reflexive thematic analysis. The multidisciplinary nature of the research team is a good starting point: please also indicate which disciplines the team represented.	We have added more information with regards to reflexivity.
**Reviewer 2**	
1. Methods: The methodology raises some questions, and requires more words to understand and trust the robustness of the methods chosen, including underlying docs (i.e. observation guides), reflexivity and ethical approval for the study (in particular since the researchers observed at departments (OR and ER) where (many?) patients and HCW's were present). See some detailed questions below.	We have rewritten the methodology section and elaborated on the data collection and analysis. In addition, we submitted a topic list for the interviews and a detailed account of our reflexive thematic analysis process in two online supplemental files. We did not use observation guides, but rather established a priori on what we wanted to focus on during observations and have mentioned these themes (i.e., observations focused on collaboration, social conventions, relationships, and atmosphere in relation to the clinical processes) in the data collection part. Moreover, we have elaborated on our reflexivity and moved the ethics approval (including our approach to establish patient consent) from the statements at the end of the manuscript to the methodology section.
2. Discussion: The authors could be more elaborate in the discussing their findings. Most of the discussion is dedicated to practice recommendations, and, although relevant, (first) critically reflecting on their own findings on a more conceptual level is a missed step. For ex. the results in terms of the undertows, raise questions for	We have reflected more on our findings using JD-R theory and literature on (post)merged health care organizations.<br><br>With respect to the specific question raised (i.e., whether the undertows could emerge even

	reflection. If they could exist independent of org practice/change, what does that to the undertows in relation to SE and QoC? In other words, could the undertows revealed emerge even when organizational change is not happening?	when organizational change was not happening), we can only speculate. Our empirical contribution was conducted in a setting where certain organizational practices (e.g., how care was organized) fundamentally changed as a result of the merger (but also COVID-19 for that matter), and as such our findings are based on that change.<br><br>Our main contribution lies in the undertows of slumbering sentiments, which do not emerge overnight, but rather emerge gradually when staff's concerns or dissatisfaction are routinely disregarded, in turn, creating more problems for their SE and QoC. Theoretically, this may also occur as a result of organizational change that is not due to mergers (e.g., COVID) or even in the absence of change.<br><br>Nevertheless, in the discussion we do reflect on the findings from literature on merged health care organizations, and in the limitations we stress how the context of this study is specific to the account produced here.
3.	The reflection should include the specific context in which the study was conducted: two departments in a post-merge hospital. Hospital merges may offer specific or additional challenges going beyond the labelling as just 'an organizational practice/ change'. There is much literature on (hospital) merges, does this study align with that lit? This may be particularly important when thinking about recommendations for practice. These may need to be more related to successful mergers than leadership or anything else. (see for ex the syst review on barriers and	Drawing from literature, we do not necessarily view merged or merging hospitals as that different from non-merging hospitals in terms of organizational change, as the latter are also subject to frequent organisational change (de Kam et al, 2020). However, we do concur that merged hospitals face intensified or long-term change (ibid), and as such warrant reflection in the discussion, which we have done with the help of the work by Fulop and co-

	facilitators to successful hospital mergers, by the Health Review Board)	authors (2005) and de Kam and colleagues (2020). We do still stand by our recommendation that leadership needs to be present and attentive, arguably even more so during periods of extensive organizational change (such as mergers).
4.	In general, this paper would improve by positioning the findings of this study in the broader literature. The authors could try better guide the reader understanding their findings in relation to i.e. theories on organizational cultures, team effectiveness and/or occupational health psychology.	We have reflected more on our findings using JD-R theory and literature on (post)merged health care organizations.
5.	Abstract: Conclusion . I'd suggest to report the main result in the first sentence, and not to repeat the question.	We have omitted the first half of the first sentence to not be repetitive.
6.	Introd<br>A: Line 121-123: Re the quote: pls explicate who said this and in what context. The fact that it is quoted raises questions. Alternatively rephrase and just keep the reference.<br><br>B: Line 124: The sentence: Empirical …..situations, needs clarification.<br><br>C: Line 126. PLs clarify what is meant by the HH paradigm. Offer the 3 pillars mentioned the explanation, or is that separate from the paradigm?<br><br>D: Main question: given the 3 pillars and aim of the study, I expect you to look at c.q. interview people about the organizational aspects of work or (line 153) organisational mechanisms in relation to the 2 pillars of SE and QoC. What is it exactly you looked for, or at, for that matter?<br>Line 153 suggests a very open perspective.<br><br>E: Line 155. Sentence seems incomplete.	A: We have paraphrased the quote.<br><br>B: We have provided a concrete example from one of the references.<br><br>C: The Healthy Healthcare Paradigm posits that all three elements (organization of care, HCW wellbeing and QoC) should be constantly considered in light of each other. We have rephrased this sentence, and as we do not elaborate on the HH paradigm anywhere else in the manuscript we chose to only reiterate their main tenet and refer to the respective authors.<br><br>D: The research project in which the data were collected examined how accountability practices impact reflection and discussion on quality. This differs from the current study's objective, which specifically examines health care worker's sustainable employability and its relationship to quality. This

	means that we were not able to specifically focus on these three elements throughout data collection. Having said that, the organizational aspects of work and care were frequently mentioned by our participants, and as such we used these more as a lens for analysis to examine these organizational aspects nonetheless. Regarding line 153, we mention the research paradigm to which we as researchers belong so readers understand why we choose our methodology (in line with the SRQR guidelines).<br><br>E: We have added more information as to which methodology we used (reflexive thematic analysis).
**7.** Line 169: Data collection:<br>A: General: Was ethical assessment obtained or waived? Pls report the evidence of at least one of them.<br><br>B: Line 175: was an interview guide used? Pls provide. Was an observational guide used? Pls provide.<br><br>C: How were the fieldnotes analysed in this process? Together with, apart from the interview data? How did you combine or integrate them?<br><br>D: Reflexivity:<br>Deserves more words. What does each person's experience and specific role (i.e. the Inspectorate), mean for the perspective they brought to the table, and how did this (potentially) impact the discussions, or even the coding?	A: We have added a separate section for ethics approval. Ethical approval was obtained after assessment.<br><br>B: A topic list was used (see one supplemental file 1); we did not use an observation guide but rather focused on prior agreed upon themes such as atmosphere and relationships.<br><br>C: Fieldnotes and interview excerpts were both coded as text in Atlas.Ti. These were not treated as separate sources in data analysis (as all of them were converted into text), but rather different data sources during collection. For a more detailed account of our data analysis process, please see online supplemental file 2.<br><br>D: We have elaborated on the different disciplines, how this multitude of backgrounds

	allowed us to 'see' more/different meanings in the data, and what that meant for data analysis.
**8.** Results<br>A: Line 217: I understand correctly you defined three themes based on the analyses.<br><br>B: Line 231-240: Various shifts are described here, not only organization ones. I assume that when the ER was closed at location X, ER personnel from this location was transferred to location Y, along with the patients. Correct?<br>Was it 'just' an organizational, logistic change, or were there also budget or personnel reductions involved? I am trying to understand whether the overburdening of personnel had other potential causes than the reorganization of practice.<br><br>C: L 255: are these italic words quotes or codes or? Italics are used throughout the results section, so I suggest the authors clarify in the first paragraph of the Results section how they should be read and understood.<br><br>D: L 257: please explain how 'readiness' and 'coordination'?<br>L 261-264: Given the phrasing it is not clear whether QoC was indeed effected by the overburdening of HCW – as expressed by them -, or that this is the authors' interpretation (QoC may become compromised).	A: We did define three themes, each referring to how a specific organisational practice contributed to a different undertow of slumbering sentiments (theme 1: through overburdening staff, theme 2: through creating an us-versus them atmosphere; theme 3: through not adequately attending to HCWs' suggestions and complaints), and how these relate to QoC and SE.<br><br>B: It is correct that ER staff from location X transferred to location Y and that the ASS unit was created to accommodate a larger influx of patients (in one ER rather than two) with limited resources (referring to a shortage of ER-nurses). Rather than needing more ER-nurses, vocationally trained nurses would be deployed at the ASS unit. Box 2 in the manuscript elaborates on the background of the development of the ASS unit.<br><br>C: These are descriptions from our participants, hence quotes. We have clarified in the introduction of the results how these should be read.<br><br>D: We have rephrased the sentence, explained what we mean with coordination of care at the ER and substituted 'readiness' with willingness. Regarding the overburdening of HCWs, the implications for compromised QoC is our interpretation though based on participants' expressed

	sentiment (i.e., reluctancy to help others) and behaviour in practice (i.e., the High Care and the ASS unit need to constantly coordinate and communicate to provide patients with adequate care).
**9.** Discussion:<br>(See general remarks above)<br><br>A: L 445: employer or employee satisfaction?<br><br>B: L 447: The examples of the reverse/positive relationship was not explicitly reported in the results. Was it explored at all? If so, consider how to include it in the reporting, in order to claim the statement made in this sentence. We can not infer that the absence of a problem self-evidently means the presence of a success.<br><br>C: L 468-478: This is an important paragraph, it interprets and elaborates on the findings, and is therefore more than a rec for practice. I'd suggest to move it up in the discussion section. The practice rec more appropriately can start at L 478 …Specifically….	A: Employer. As in, reducing the satisfaction of employees towards their job and employers. We have rephrased to clarify.<br><br>B: As our study participants did not (report to) feel heard, valued or supported during the merger, we were also not able to report any positive effects for their wellbeing. However, we did want to mention that – according to the work by Gordon et al. (2022) - had there been appropriate support and attention for employees during disruptive events (such as mergers) positive consequences to their wellbeing may have been possible.<br><br>9C: We have followed up on your suggestion and moved the first few sentences from the practice recommendations to the second paragraph of our discussion (referring to the main contribution and message).
**10.** A conclusion is missing. Pls add.	We have added a conclusion
**Additional changes by authors in revised manuscript**	
Changed bulletpoints strengths and limitations after abstract	In line with the methodological comments and suggestions made by reviewer 1, we also reformulated our strengths and limitations for this study after the abstract.
Added reference of recently published study	Since our submission to BMJ Open, our umbrella review on the relationship between SE of HCWS and QoC has been published. Given its relevance to

	this study, we have added this study as a reference in the introduction
In line with suggestions from the Reflexive Thematic Analysis Reporting Guidelines by Braun & Clarke, we have changed some headings	- Methods > Methodology<br>- Data collection > Data generation<br>- Results > Analysis<br>- Strengths and limitations > Evaluating our approach

VERSION 2 - REVIEW

Reviewer 1

Name Juvonen-Posti, Pirjo

Affiliation Finnish Institute of Occupational Health

Date 23-Nov-2025

COI

Thank you for the opportunity to re-evaluate the article! The authors responded to the correction requests accurately and to a sufficient extent. In their correction process, they realized some key things about the method they used and accordingly made major corrections to the text of the article. I was delighted with the description of the results the first time around, now opening the analysis process has reinforced my impression. I found only one typo on line 649 "oranganisation". I recommend accepting the article.
